# Time Changes of Survival and Cardiovascular Determinants in a Cohort of Middle-Aged Men Followed Up for 61 Years until Extinction

**DOI:** 10.3390/jcdd11070221

**Published:** 2024-07-13

**Authors:** Alessandro Menotti, Paolo Emilio Puddu

**Affiliations:** 1 Association for Cardiac Research, Via Voghera, 31, 00182 Rome, Italy; amenotti2@gmail.com; 2 EA 4650, Signalisation, Électrophysiologie et Imagerie des Lésions D’ischémie Reperfusion Myocardique, Université de Normandie, 14000 Caen, France

**Keywords:** survival, long-term follow-up, middle-aged men, personal characteristics, lifestyle behaviors, chronic diseases

## Abstract

Objective: To study possible determinants of longevity in a cohort of middle-aged men followed for 61 years until extinction using measurements taken at baseline and at years 31 or 61 of follow-up. Material and Methods: In 1960, two rural cohorts including a total of 1712 men aged 40–59 years were enrolled within the Italian section of the Seven Countries Study of Cardiovascular Diseases, and measurements related to mainly cardiovascular risk factors, lifestyle behaviors, and chronic diseases were taken at year 0 and year 31 of follow-up (when only 390 could be examined). Multiple linear regression models were computed to relate personal characteristics with the length of survival in both dead men and survivors. Results: Baseline cardiovascular risk factors, smoking and dietary habits, and chronic diseases (taken at year 0 with men aged 40–59 years) were significant predictors of the length of survival both from year 0 to year 31 and from year 0 to year 61, but only chronic diseases were independent predictors for the period of 31 to 61 years. Significant predictors of survival using measurements taken at year 31 (age range 71 to 90 years) were only smoking and dietary habits and chronic diseases. Conclusions: During a lifetime of follow-up, the personal characteristics with continuous predictive power of survival were only lifestyle behaviors and major chronic diseases.

## 1. Introduction

Two rural cohorts of middle-aged men enrolled in Italy within the Seven Countries Study of Cardiovascular Diseases were examined in 1960 and followed up for 61 years until extinction. A comprehensive analysis on age at death as a function of a large number of personal characteristics measured at entry examination has already been reported in detail [1]. However, it was not clear for how long those possible determinants of longevity were active in their role of predictors, although the largest proportion among them involved cardiovascular origin, like systolic blood pressure, cholesterol levels, heart rate, and dietary and motion habits [1].

The availability of another comprehensive field re-examination performed around mid-way through the entire follow-up (year 31) prompted the idea to use it, beyond the one performed at baseline [1], to study the changing role of possible determinants of survival during the entire lifespan of the study population. Although we could not find studies fully comparable to the present one, or only just similar in terms of variables measured (especially of cardiovascular nature), length of follow-up, and repetition of assessments in the existing literature, it was speculated that the results obtained at the mid-point of an extremely long observation period in a residential cohort of middle-aged men might provide clues to understand which are the factors most heavily involved in longevity and survival, thus justifying the present report. Indeed, most of the potential determinants of longevity available for this analysis are traditional cardiovascular risk factors and lifestyle behaviors that are frequently predictive of major cardiovascular diseases together with specific major nutrients. Moreover, some selected prevalent cardiovascular diagnosed conditions are part of the explored possible determinants of longevity together with some other major diseases such as chronic bronchitis, diabetes, and cancer [1].

The literature is actually full of papers claiming the existence of a long list of determinants of longevity, but certain contributions, including the long-term follow-up of the study population until old age or extinction are extremely rare, and only a few might be considered [2,3,4,5,6,7], including another one that proposes innovative procedures for predicting longevity [8]. All of these papers [2,3,4,5,6,7,8] are characterized by subsequent temporal steps, including baseline measurements and relatively long follow-up, allowing for the computation of differential survival as a function of risk factors and other personal characteristics, similar to the aim of the present study.

## 2. Material and Methods

### 2.1. Population and Measurements

In 1960, two rural cohorts of middle-aged men (entry age: 40 to 59 years, participation rate: 98.7%) were enrolled and examined within the Italian section of the Seven Countries Study of Cardiovascular Diseases (SCS). They represented men aged 40 to 59 years old residing in a rural community in Northern Italy (Region of Emilia-Romagna) and another one in Central Italy (Region of Marche). About two-thirds of the men were farmers engaged in heavy physical activity in the middle of last century, when agriculture was mechanized very little. Baseline examination measured a number of personal characteristics, including social, behavioral, anthropometric, biophysical, biochemical, and clinical data [9]. During the first 31 years of follow-up, out of the initial 1712 subjects, 1090 had died and 622 had survived, reaching the age range of 71–90 years. At year 31, a subgroup of 390 men was re-examined, representing only 63% of the survivors. This group is now one of the main objects of the present analysis.

In the analysis, measurements taken at year 0 and year 31 of follow-up were used only if available at both points, as follows: (a) age, approximated to the nearest birthday (years); (b) body mass index (kg/m^2^), subscapular skinfold (mm), and systolic blood pressure (mmHg), measured following the rules given in the WHO Cardiovascular Survey Methods manual [10]; (c) smoking habits (never, ex-, and current smokers), derived from a questionnaire; (d) heart rate, measured on a resting electrocardiogram (ECG) (beats/min); (e) serum cholesterol (mg/dL), measured by the Anderson, Keys technique in 1960 [11] and the Allain et al. technique in 1991 [12]; (f) ECG abnormalities, defined by the presence of any ECG Minnesota Code, edition 1968 [10], corresponding to major Q waves, negative T waves, type 1 atrioventricular block, left bundle branch block, intraventricular block, or atrial fibrillation (all coded: yes, no); (g) prevalent diseases defined by SCS criteria [13]: coronary heart disease (CHD), heart failure, stroke, peripheral artery diseases, cancer, diabetes, or chronic bronchitis (all coded: yes, no); (h) in both examinations, dietary surveys were run with the dietary history method using a questionnaire administered by trained and supervised dietitians.

A group of 18 food groups were identified, and local (Italian) food tables [14,15] were used to convert the questionnaire findings into major nutrients. Among the available nutrients, we used protein, saturated fatty acids (SAFA), monounsaturated fatty acids (MUFA), polyunsaturated fatty acids (PUFA), protein, oligosaccharides, and polysaccharides. The 18 food groups were also used to construct a dietary score by choosing one out of four factor scores derived from a principal component analysis, then subdivided into three tertiles arbitrarily called the following: 1 = Unhealthy Diet; 2 = Intermediate Diet; and 3 = Healthy Diet. The trend across the three arbitrary classes of this a-posteriori score was validated by computing the mean levels, for each of them, of the natural logarithm of the Mediterranean Adequacy Index (lnMAI) [16], an a-priori dietary score created on the basis of the Mediterranean Diet. Healthy Diet had a higher level than Intermediate and Unhealthy Diets.

The food groups used for the above purpose were bread, cereals, potatoes, legumes, vegetables, fruit, oils, meat, fish, eggs, butter, margarine, milk, cheese, sugar, pastries, alcohol, and sugar beverages. More details can be found elsewhere [9,17].

### 2.2. Statistical Analysis

Distributions of variables were computed and reported as means and standard deviations for continuous variables and percent proportions and standard error for discrete variables separately for the measurements at year 0 (on 1712 men) and at year 31 (on 390 men).

Survival (SUR) was computed in years for both survivors and the deceased from the date of examination and measurement of determinants (years 0 or 31), and the deadlines were year 31 and year 61 of follow-up. A preliminary analysis was conducted which compared some baseline measurements and the final outcome between the group of 390 men examined at year 31 and the 232 men that did not attend the 31-year examination.

Multiple linear regression (MLR) models were computed with SUR as the dependent variable and several possible determinants as independent variables, as follows: (A) Model 1: baseline (year 0) measurements predicting length of SUR from year 0 to year 61 in all men; (B) Model 2: baseline (year 0) measurements predicting length of SUR from year 0 to year 31 in all men; (C) Model 3: baseline (year 0) measurements predicting length of SUR from year 31 to year 61 in men alive at year 31; (D) Model 4: baseline (year 0) measurements predicting length of SUR from year 31 to year 61 in men examined at year 31; (E) Model 5: measurements taken at year 31 predicting length of SUR from year 31 to year 61 in men examined at year 31.

Using the model (A) predicting survival from year 0 to year 61 and, separately, the one predicting survival from year 31 to year 61 (with measurements taken at year 31), theorical estimates of length of survival were made by adopting arbitrary choices of modifiable risk factor levels after having excluded the role of the others (mainly prevalent diseases) using the proper procedures.

## 3. Results

During the 61 years of follow-up, 1708 men out of the 1712 enrolled at baseline had died; 3 were still alive, with ages ranging from 102 to 106 years; and 1 was lost to follow-up after 50 years, when he was aged 91 years.

Panel 1 of Table 1 is a demographic description of what happened during the first 31 years and subsequently. The most instructive finding is the advantage of men reaching year 31 of follow-up alive, since their average outlook was to reach the age of 86 years, while the average expectation at year 0 was to reach 75–76 years of age. Those who died before year 31 had a mean survival of 22 years.

The mean levels of the variables used in the analysis related to the entry examination of year 0 (on 1712 subjects) and year 31 (on 390 subjects) are reported in Panel 2 of Table 1. Overall, there was a large and significant increase in the levels of almost all variables, except never smokers and heart rate, while a decrease was seen for current smokers. The estimates of the three types of diet could not be directly evaluated since they referred to relative values arbitrarily defined in tertile groups from the factor scores of two independent principal component analyses. However, the profile derived from the food groups and the nutrients showed a tendency of the dietary habits to be less healthy at year 31 than at year 0. In particular, there was an improvement in the direction towards a healthy diet for three nutrients, but a worsening for another three, as well as an improvement for six food groups and a worsening for another nine food groups. Other favorable trends were a reduction in energy intake and a sharp reduction in alcohol intake. Altogether, the net result based on the overall change in the lnMAI [16] was a worsening (decrease) of this indicator of the Mediterranean Diet, although the trend across the three levels was roughly maintained.

The evaluation of personal characteristics at year 31 was hampered by a low participation rate in the examination performed on the survivors, with 622 alive and 390 examined (63%). A systematic comparison of entry characteristics between the two groups (390 examined versus 232 not examined) showed a few significant differences, i.e., the non-examined subjects had higher ages by 1.5 years, slightly higher levels of serum cholesterol, slightly lower forced expiratory volumes, and a lower prevalence of chronic bronchitis. In the long run, the distribution of 26 groups of causes of death were not different between the two groups, but the non-examined had an age at death 1 year lower.

Table 2, Table 3, Table 4, Table 5 and Table 6 report the findings of the MLR models. Models 1 and 2, based on the possible predictive role of baseline measurements (Table 2 and Table 3) on 1712 men seen at year 0, cover the periods from 0 to 61 and from 0 to 31 years. The findings were relatively similar, with beneficial roles of never smokers, dietary score, and PUFA and adverse roles of age, blood pressure, heart rate, serum cholesterol, SAFA, stroke, cancer, diabetes, chronic bronchitis, and the addition of subscapular skinfold (inverse relationship) in the 0 to 31 years follow-up model. The R^2^ (the square of the linear correlation coefficient, explaining the proportion explained) of the models had satisfactory levels of 0.30 and 0.23, respectively. Models 3 and 4 (Table 4 and Table 5), still based on entry risk factor levels, dealt with all survivors at year 31 and, separately, the portion of them examined at the same time. In both cases, some baseline prevalent diseases could not be used, since all those carrying them were already dead at the beginning of the follow-up period from year 31 to year 61. In general, the majority of risk factors lost their significant predictive roles, with only ex- and never smokers showing protective effects in both models, cholesterol and diabetes showing adverse effects in Model 3, and systolic blood pressure showing an adverse effect in Model 4. Despite this, the R^2^ of the models had satisfactory levels of 0.23 and 0.24. Model 5 (Table 6), dealing with men examined at year 31 and risk factors measured at that point, also provided a few significant risk factors, i.e., the direct role of ex- and never smokers and of healthy diet and the inverse role of silent ECG findings and of three prevalent morbid conditions (peripheral artery disease, stroke, and diabetes). The R^2^ was 0.32.

Comparative inspection of the five MLR models is difficult; therefore, a simplified summary tabulation is given in Table 7. A direct association of never smokers was found on five occasions; for ex-smokers and dietary score, on three occasions; and for PUFA, on two occasions. Among the determinants playing adverse roles, age was found five times; systolic blood pressure was found three times together with cholesterol, stroke, and diabetes; and SAFA, cancer, chronic bronchitis, and heart rate were each found twice. In general, during the last years of follow-up, some risk factors tended to lose the predictive power of survival, partly substituted by the prevalent chronic diseases.

Each of the five MLR models displayed in Table 2, Table 3, Table 4, Table 5 and Table 6 carry relatively high levels of R^2^ ranging from 0.23 to 0.32, corresponding to Rs of 0.48 to 0.57. The most important comparison was that predicting survival in men examined at year 31 using measurements taken at baseline (year 0) (R = 0.49) versus measurements taken at year 31 (R = 0.57). The latter was larger, but the difference was not statistically significant. However, in both models, a prevalent and significant predictive role was played by morbid conditions. In the models using the 31-year measurements, smoking and dietary habits were also significant predictors.

A way to look at the practical consequences of the above models may consist of estimating, in some examples, the predicted length of survival based on potentially significant and modifiable risk factor levels, starting at year 0 and year 31 and using the respective coefficients of the multivariate models. The numbers reported in Table 8 are arbitrary choices among an almost infinite number of combinations. The length of survival was estimated by using the average risk factor levels and then by using better and worse risk factor profiles roughly corresponding to differences of plus or minus one standard deviation for continuous variables and different choices for discrete variables.

After forcing the average population levels into the models, the outcomes refer only to groups of individuals showing those mean levels. On the other hand, when one makes estimates for a single individual, clear choices can also be made for discrete variables. A satisfactory prolongation of survival was found for good risk factor profiles in both models. However, there were larger prolongations in the model of 0 to 61 years than in the one of 31 to 61 years. This effect can be attributed to the available lengths of survival, which were largely different between the two models, starting from average ages of 49.1 and 77.4 years, respectively.

Actually, the percent gain in survival moving from an average to a good risk factor profile was 23.5% for the estimate of 0 to 61 years and somewhat higher, i.e., 27.8%, for the estimate of 31 to 61 years. This seems to be a great outcome. However, one should be cautious when considering the lower significance of coefficients in the model of 31 to 61 years than in that of 0 to 61 years. The reverse outcome, that is, moving from the average to the bad risk factor profile, provided a loss of survival of 7.4 years for the model of 0 to 61 years and 3.8 year for the model of 31 to 61 years, corresponding to losses of 28% and 37.6%, respectively.

The aforementioned estimates do not take into account the additional role of prevalent diseases, the impact of which can be roughly estimated by algebraically adding their multivariate coefficients. In this case, the lost years were very high for prevalent diseases at baseline (mainly cancer, with 19.5 years; stroke, with 11.7 years; CHD, with 4.9 years (not significant); and chronic bronchitis, with 3.5 years) versus losses for prevalent cases at year 31 (stroke, with 2.6 years; peripheral artery disease, with 2.3 years; diabetes, with 1.9 years; and major silent ECG abnormalities, with 3.2 years). Again, the available time in front of the examinees was much longer starting at year 0 than starting at year 31. Moreover, all those carrying any one of CHD, stroke, heart failure, or cancer at baseline were dead after the first 31 years of follow-up, and these variables could not be used for prediction of survival for the period of 31 to 61 years.

With simple personal computer programs, it is possible to replicate these estimates for any combination of risk factors.

## 4. Discussion

This analysis is unique in the cardiovascular epidemiological field, since the individuals were derived from residential cohorts of middle-aged men, the personal characteristics were rather numerous, and two examinations were held at year 0 and year 31 of a follow-up reaching 61 years and the extinction of the cohorts. Prediction of survival as a function of the measured characteristics was allowed, and the time changes in the predicting power of these characteristics were investigated and compared between the two periods. In particular, estimates could be made for men aged 40–59 years during a follow-up of 61 years and again on the survivors after 31 years, who were then aged 71–90 years, with the time horizon of the next 30 years.

Although it is known that some risk factors, lifestyle behaviors, and other personal characteristics can be determinants of longevity, here, we had the ability to use all of them together in this analysis. Table 2, Table 3, Table 4, Table 5 and Table 6 and the simplified summary of Table 7 enabled two distinct survival predictions to be performed in men with average ages of 49.1 and 77.4 years, respectively. The practical implications were those of Table 8, where good versus bad risk factor profiles are directly compared. The actual number of years gained or lost may thus be appreciated by applying the reported MLR results of this investigation. This has no similar counterpart in the present literature.

A tremendous change in medical practice and prevention occurred in recent years, to the extent that the habits, actions, and treatments of 60 years ago are certainly not comparable to the present. However, in case one wishes to have a long-term outcome result regarding what is performed today, we should wait half a century at least. This reinforces the present results for projective and preventive measures in the near future, while waiting is necessary in order to obtain the long-term impacts of the present attitudes.

In the present investigation, several individual characteristics could be used, but there was at least one important limitation since, wishing to compare two periods, we excluded the classification of physical activity as it was not available at the year 31 examination. In fact, the baseline measurement showed, in previous analyses, the strong predictive and protective power of vigorous physical activity versus age at death and other end-points [1].

Overall, the available risk factors and lifestyle behaviors, like smoking habits and dietary habits, measured at year 0 were significantly predictive of survival to years 31 and 61. This was not the case for those measured at year 31 for predictive purposes to year 61, with the exception of the continuous favorable role of being a never smoker and, partly, of the adverse role of high blood pressure. Incidentally, for some risk factors, the coefficients for the period of 0 to 61 years roughly corresponded to the sum of those found for the periods of year 0 to 31 and year 31 to 61, although the latter were not significant.

The role of prevalent major chronic diseases has always been heavy, contributing to a reduction in survival of several years. We noticed a strange exception since, for unclear reasons, the predictive role of CHD was never significant despite the production of relatively large multivariate coefficients. Another problem was the insignificant role of prevalent cancer among those examined at year 31. We tested the cancer death rates of the examined versus the non-examined patients, but the difference was not significant, although it was slightly larger among the non-examined. However, this kind of exception is part of the expected outcome when the model includes a large number of covariates (around 20 or more) and the baseline numbers of the statistical units are small.

Other limitations of this analysis are the small size of the entry (and subsequent) denominators, the availability of only male subjects, and the limited number and types of risk factors measured in both examinations, not including more recent markers that could not be used more than 60 years ago. Another apparent limitation is due to the large age range of men enrolled at entry (40 to 59 years), but the multivariate coefficient of age (present in all MLR models and always negative) directly indicates the number of years lost for a 1-year difference in entry age.

The search for useful references for comparison with our analysis was very difficult. For example, PubMed, under the request “early determinants of longevity”, provides thousands of papers, the absolute majority of which are definitely not comparable with our data and analysis. After exploring 1000 papers, an incredible list of bio-medical problems was individually presented (almost never in combinations), and whenever they proved to be beneficial or dangerous for human health, they were defined and classified as beneficial or dangerous factors in terms of longevity. Moreover, systematic measurements taken on cohorts of individuals then followed up for long enough to evaluate them as real determinants of longevity are extremely rare. Actually, half of the references were related to animal (or even vegetable) longevity, starting from elephants and progressing down to cows, rats, nematodes, rare butterflies living two days, *Drosophila melanogaster*, and many more. Among the many proposed determinants of longevity, there were social relations [18], chronic disease risk factors [19], a theoretical estimate of chronic disease decrease [20], serum cholesterol [21], heart rate variability [22], caloric restriction [23], size at birth [24], metabolic rate [25], obesity [26], elevated B-type natriuretic peptide [27], adverse role of undernutrition in pregnant women [28], and ancestors’ nutrition [29]. The problem is that they were treated one by one, frequently in a “narrative” format, and almost never with a direct measurement followed by a proper follow-up until a real old age was reached. Another example of the peculiar literature on this issue is exemplified by a book dealing with Japanese centenarians [30], where everything is described in terms of how they are at that age, but nothing can be found on how they were when they were younger at any given starting point, nor which characteristics could thus predict their long life expectancies. However, some interesting contributions could be found. For example, a comparison was made using local risk functions and specific causes of death to estimate the roles of various risk factors in longevity, starting from the age of 40 [31]. Cigarette smoking, high blood pressure, physical inactivity, high blood glucose, high dietary salt intake, and alcohol use were, in decreasing order, the most important predictors limiting survival. In a review paper comparing data from the G7 countries, Japan had the longest life expectancy. High death rates from stroke and stomach cancer have recently decreased, while the low rates form coronary heart disease have been attributed to a basic plant food diet, high fish intake, and a reduction in salt consumption. At the same time, some Western dietary habits are still rare [32].

A few contributions had real similarities to our analysis, and some of them deserve quotation. In Australia, a study of more than 12,000 women aged 70–75 who were followed up for 20 years showed that the subgroup identified as successful agers (5.5%) was characterized at baseline by no major diseases or disabilities, low body mass index, no smoking, high education, and good social support [2]. In a Swedish study run on more than 1000 men and women aged 70 years, the use of a refined version of the Mediterranean Diet was associated with subsequent lower mortality, with an additional contribution of being a non-smoker and of low levels of the usual cardiovascular risk factors [3]. A recent important contribution regarding the role of diet was published by the Willett research group, finding that healthy dietary habits may prevent various chronic diseases, although this does not demonstrate the consequences on the age of the participants at death [33]. In the British Physicians’ Health Study, 2357 men with a mean age of 72 years were followed for at least 16 years [4]. Major risk factors for mortality were smoking habits, diabetes, obesity, and hypertension, while regular exercise and lower incidence of chronic diseases were associated with lower mortality. In a Dutch study on 2829 middle-aged civil servants and their spouses, who were followed up for 25 years, the use of a prudent diet (characterized by higher intake of brown bread, porridge, yogurt, vegetables, fish, and fruit) was associated with lower mortality in men, but this effect was not found among women [5]. In a small group of subjects aged 80–98 years, everyday physical activity was evaluated by mechanical actigraphs, and in a short follow-up period of 2 years, sedentary people had a death rate three times higher than those classified as active [6]. In a classic study on over 16,000 Harvard alumni aged 35 to 75 years, all-cause mortality was evaluated during 12 to 16 years [7]. Death rates declined with increasing kcal spent per week on various physical activities. By the age of 80 years, survival attributable to adequate exercise was 1 to 2 years longer compared to sedentariness.

A special contribution needs mentioning, since it deals with an innovative approach [8] consisting of the evaluation of biological aging in 900 young adults through the measurement of multiple organ deterioration. This will allow for a definition of the biological age and, perhaps, predicts the occurrence of major diseases and the length of survival. However, the final proof of the value of this complex approach will come only if the follow-up, which has already started, is extended until old ages and possibly until the extinction of the cohort, which is still a long way away.

Herein, we did not consider nor discuss genetic problems that were outside of our possibilities in the original investigation started in the 1960s. However, we would like to quote one of the many papers dealing with the association of telomere length with longevity. In a group of Ashkenazi Jewish centenarians, it was found that they and their siblings maintained their telomere length compared with controls and remained free from major chronic diseases [34].

There have been several articles based on large demographic data, some of which are of interest. The Mediterranean Island of Sardinia, in Italy, is renowned for its alleged high rate of centenarians. A recent contribution stated that the phenomenon is localized only in certain areas and seems not to be associated with a special diet, while it seems apparently related to the hilly terrain of their country and the need to walk up and down steep slopes, leading to significant energy expenditure [35]. In over 800,000 respondents to the Cancer Prevention Study in the USA, it was clearly shown that, when smoking cessation occurred at younger ages, there was greater benefit in terms of life extension, but years of life could be gained even when cessation occurred late in life [36]. Data derived from the population censuses of 1900 and 1930 in the USA showed that parental longevity and some midlife characteristics were significant predictors of longevity, together with being born in the second half of the year. Moreover, the wives of male centenarians had significantly better survival compared to the wives of centenarians’ brothers [37].

## 5. Conclusions

A small set of cardiovascular risk factors, some lifestyle behaviors, and the prevalence of major chronic diseases measured in cohorts of middle-aged men were associated with prediction of survival until the extinction of the original population after 61 years of follow-up. Similar measurements taken in survivors after 31 years were also predictive, but a major role in the late period of life was played by lifestyle behaviors, such as smoking and dietary habits and the prevalence of chronic diseases.

## Figures and Tables

**Table 1:** Demographic picture and mean levels of risk factors measured in all men at year 0 (n = 1712) and in those attending the 31-year re-examination (n = 390). Significant risk factors in **Bold**.

Panel 1. DEMOGRAPHIC PICTURE.							
	n	Age	Dead in 31 Years	Dead in 61 Years	Years of Survival in Year 0 to 31	Years of Survival in Year 0 to 61	Years of Survival in Year 31 to 61
Examined in year 0	1712	49.1 (5.1)	1090	1708	22.6 (8.6)	26.4 (12.9)	---
Alive in year 31	622	77.4 (4.2)	---	618	---	---	9.3 (6.2)
Examined in year 31	390	77.4 (4.1)	---	389	---	---	10.1 (6.2)
**Panel 2. RISK FACTORS**							
			**Year 0: n = 1712**		**Year 31: n = 390**		***p* of difference**
**Risk factor**			**mean**	SD (*)	**Mean**	SD (*)	
**Age, years**			**49.1**	**5.1**	**77.4**	**4.1**	**<0.0001**
**Smokers, %**			**61.1**	**1.2**	**15.9**	**1.9**	**<0.0001**
**Ex-smokers, %**			**13.6**	**0.8**	**61.8**	**2.5**	**<0.0001**
Never smokers, %			25.4	1.1	22.3	2.1	0.20
Unhealthy diet, %			33.3	47.2	33.3	47.2	---
Intermediate diet, %			33.4	47.2	33.4	47.2	---
Healthy diet, %			33.3	47.2	33.3	47.2	---
**Body mass index, kg/m^2^**			**25.2**	**3.7**	**26.1**	**3.5**	**<0.0001**
**Subscapular skinfold, mm**			**11.8**	**5.9**	**19.3**	**6.4**	**<0.0001**
**Systolic blood pressure, mmHg**			**143.6**	**21.0**	**160.5**	**19.6**	**<0.0001**
Heart rate, beats/min			71.3	12.9	70.3	13.5	0.19
**Serum cholesterol, mg/dL**			**201.6**	**40.8**	**208.2**	**41.0**	**0.004**
Protein, gr/day			76.5	24.5	76.1	20.9	0.77
**SAFA, gday**			**28.7**	**11.7**	**20.6**	**8.8**	**<0.0001**
**MUFA, g/day**			**49.3**	**16.3**	**47.3**	**15.3**	**0.027**
**PUFA, g/day**			**11.4**	**6.8**	**8.1**	**5.4**	**<0.0001**
**Polysaccharides, g/day**			**254.7**	**90.8**	**200.3**	**67.4**	**<0.0001**
**Oligosaccharides, g/day**			**57.9**	**34.5**	**74.1**	**31.3**	**<0.0001**
**Myocardial infarction, %**			**1.1**	**0.3**	**7.4**	**1.3**	**<0.0001**
**Heart failure, %**			**0.9**	**0.2**	**26.7**	**2.2**	**<0.0001**
**Stroke, %**			**0.8**	**0.2**	**9.5**	**1.5**	**<0.0001**
**Peripheral artery disease, %**			**0.4**	**0.2**	**20.5**	**2.0**	**<0.0001**
**Cancer, %**			**0.3**	**0.1**	**6.7**	**1.3**	**<0.0001**
**Diabetes, %**			**4.7**	**0.5**	**13.6**	**1.7**	**<0.0001**
**Chronic bronchitis, %**			**6.3**	**0.6**	**41.3**	**2.5**	**<0.0001**
**ECG abnormal silent, %**			**1.9**	**0.3**	**9.0**	**1.4**	**<0.0001**
**ECG abnormal in heart disease, %**			**1.0**	**0.2**	**6.2**	**1.2**	**<0.0001**

(*) Standard error for discrete variables expressed in %.

**Table 2:** MLR Model 1 with covariates measured at baseline in 1712 men, with length of survival after 61 years of follow-up as the dependent variable. R^2^ = 0.30. Significant risk factors in **Bold**.

Risk Factor	Coefficient	*p* of Coefficient	Delta	Gained (+) or Lost (−) Years	lcl	hcl
**Intercept**	**9.13**	**<0.0001**	**1**	**---**	**---**	**---**
**Age, years**	**−0.93**	**<0.0001**	**5**	**−4.64**	**−5.19**	**−4.10**
Smokers, %	reference	---	---	---	---	---
Ex-smokers, %	1.46	0.074	1	1.46	−0.14	3.05
**Never smokers, %**	**3.15**	**<0.0001**	**1**	**3.15**	**1.89**	**4.40**
Unhealthy diet, %	reference	---	---	---	---	---
Intermediate diet, %	1.29	0.059	1	1.29	−0.05	2.63
**Healthy diet, %**	**2.072**	**0.007**	**1**	**2.07**	**0.57**	**3.57**
Body mass index, kg/m^2^	0.082	0.48	3.5	0.29	−0.51	1.08
Subscapular skinfold, mm	0.13	0.082	6.0	0.76	−0.09	1.62
**Systolic blood pressure, mmHg**	**−0.13**	**<0.0001**	**20**	**−2.33**	**−2.91**	**−1.75**
**Heart rate, beats/min**	**−0.057**	**0.01**	**13**	**−0.74**	**−1.31**	**−0.18**
**Serum cholesterol, mg/dL**	**−0.027**	**0.0001**	**40**	**−1.07**	**−1.60**	**−0.54**
Protein, gr/day	−0.002	0.92	25	−0.04	−0.93	0.84
**SAFA, gr/day**	**−0.077**	**0.048**	**12**	**−0.92**	**−1.84**	**−0.01**
MUFA, gr/day	0.034	0.12	16	0.54	−0.14	1.22
**PUFA, gr/day**	**0.087**	**0.043**	**7**	**0.61**	**0.03**	**1.19**
Polysaccharides, gr/day	−0.001	0.73	90	−0.12	−0.80	0.56
Oligosaccharides, gr/day	0.017	0.075	35	0.61	−0.06	1.28
Myocardial infarction, %	−4.88	0.17	1	−4.88	−11.81	2.04
Heart failure, %	0.54	0.86	1	0.54	−5.560	6.58
**Stroke, %**	**−11.66**	**0.0001**	**1**	**−11.66**	**−17.64**	**−5.68**
Peripheral artery disease, %	1.099	0.79	1	1.10	−7.06	9.26
**Cancer, %**	**−19.52**	**0.0001**	**1**	**−19.52**	**−29.12**	**−9.92**
**Diabetes, %**	**−2.97**	**0.019**	**1**	**−2.97**	**−5.44**	**−0.50**
**Chronic bronchitis, %**	**−3.46**	**0.002**	**1**	**−3.46**	**−5.62**	**−1.30**
ECG abnormal silent, %	−1.98	0.31	1	−1.98	−5.84	1.87
ECG abnormal in heart disease, %	−4.57	0.24	1	−4.57	−12.22	3.09

*p* of coefficient = probabilities. Delta = difference in the levels of risk factors for the estimate of gained or lost years. lcI: low 95% confidence interval; hcI: high 95% confidence interval.

**Table 3:** MLR Model 2, with covariates measured at baseline in 1712 men and length of survival after 31 years of follow-up as the dependent variable. R^2^ = 0.23. Significant risk factors in **Bold**.

Risk Factor	Coefficient	*p* of Coefficient	Delta	Gained (+) or Lost (−) Years	lcl	hcl
Intercept	57.90	---	---	---	---	---
**Age, years**	**−0.45**	**<0.0001**	**5**	**−2.26**	**−2.64**	**−1.89**
Smokers, %	reference					
Ex-smokers, %	0.049	0.93	1	0.05	−1.06	1.16
**Never smokers, %**	**1.26**	**0.006**	**1**	**1.26**	**0.36**	**2.10**
Unhealthy diet, %	0.65	0.17	1	0.65	−0.29	1.58
**Intermediate diet, %**	**1.21**	**0.023**	**1**	**1.21**	**0.17**	**2.25**
Healthy diet, %	0.076	0.35	3.5	0.27	−0.29	0.82
**Body mass index, kg/m^2^**	**0.10**	**0.04**	**6.0**	**0.62**	**0.02**	**1.22**
**Subscapular skinfold, mm**	**−0.083**	**<0.0001**	**20**	**−1.65**	**−2.06**	**−1.25**
**Systolic blood pressure, mmHg**	**−0.047**	**0.003**	**13**	**−0.61**	**−1.00**	**−0.21**
**Heart rate, beats/min**	**−0.013**	**0.008**	**40**	**−0.51**	**−0.88**	**−0.14**
Serum cholesterol, mg/dL	0.003	0.80	25.0	0.08	−0.54	0.70
**Protein, gr/day**	**−0.063**	**0.019**	**12.0**	**−0.76**	**−1.40**	**−0.12**
SAFA, gr/day	0.026	0.092	16.0	0.41	−0.07	0.88
**MUFA, gr/day**	**0.058**	**0.029**	**7.0**	**0.40**	**0.00**	**0.81**
PUFA, gr/day	−0.0001	0.98	90.0	−0.01	−0.48	0.46
Polysaccharides, gr/day	0.013	0.053	35.0	0.46	−0.01	0.93
Oligosaccharides, gr/day	−2.66	0.28	1	−2.66	−7.48	2.16
Myocardial infarction, %	0.53	0.81	1	0.53	−3.67	4.73
**Heart failure, %**	**−9.61**	**<0.0001**	**1**	**−9.61**	**−13.77**	**−5.45**
Stroke, %	0.79	0.79	1	0.79	−4.89	6.46
**Peripheral artery disease, %**	**−15.55**	**<0.0001**	**1**	**−15.55**	**−22.22**	**−8.87**
Cancer, %	−1.46	0.096	1	−1.46	3.18	0.26
**Diabetes, %**	**−2.72**	**0.0004**	**1**	**−2.72**	**−4.23**	**−1.22**
Chronic bronchitis, %	−1.22	0.37	1	−1.22	−3.90	1.46
ECG abnormal silent, %	−4.94	0.069	1	−4.94	−10.26	0.39

*p* of coefficient = probabilities. Delta = difference in the levels of risk factors for the estimate of gained or lost years. lcI: low 95% confidence interval; hcI: high 95% confidence interval.

**Table 4:** MLR Model 3, with covariates measured at baseline in 622 men alive at year 31 versus length of survival from year 31 to 61 of follow-up as the dependent variable. R^2^ = 0.23. Significant risk factors in **Bold**.

Risk Factor	Coefficient	*p* of Coefficient	Delta	Gained (+) or Lost (−) Years	lcl	hcl
Intercept	69.58	---	---	---	---	---
**Age, years**	**−0.59**	**<0.0001**	**5**	**−2.94**	**−3.47**	**−2.41**
Smokers, %	reference	---	---	---	---	---
**Ex-smokers, %**	**1.47**	**0.029**	**1**	**1.47**	**0.16**	**2.78**
**Never smokers, %**	**2.64**	**<0.0001**	**1**	**2.64**	**1.64**	**3.64**
Unhealthy diet, %	reference	---	---	---	---	---
Intermediate diet, %	1.12	0.074	1	1.12	−0.11	2.34
Healthy diet, %	0.78	0.27	1	0.78	−0.62	2.19
Body mass index, kg/m^2^	0.16	0.14	3.5	0.55	−0.18	1.29
Subscapular skinfold, mm	−0.038	0.55	6.0	−0.22	−0.96	0.51
Systolic blood pressure, mmHg	−0.031	0.059	20	−0.63	−1.28	0.02
Heart rate, beats/min	0.010	0.64	13	0.13	−0.43	0.69
**Serum cholesterol, mg/dL**	**−0.015**	**0.016**	**40**	**−0.58**	**−1.05**	**−0.11**
Protein, gr/day	0.001	0.93	25.0	0.03	−0.67	0.73
SAFA, gr/day	−0.054	0.15	12.0	−0.58	−1.38	0.22
MUFA, gr/day	0.011	0.57	16.0	0.17	−0.43	0.78
PUFA, gr/day	0.051	0.17	7.0	0.36	−0.15	0.87
Polysaccharides, gr/day	−0.004	0.16	90.0	−0.39	−0.94	0.15
Oligosaccharides, gr/day	0.009	0.28	35.0	0.30	−0.25	0.86
Myocardial infarction, %	excluded	---	1	---	---	---
Heart failure, %	excluded	---	1	---	---	---
Stroke, %	excluded	---	1	---	---	---
Peripheral artery disease, %	excluded	---	1	---	---	---
Cancer, %	−1.081	0.74	1	−1.08	−7.48	5.32
**Diabetes, %**	**−3.65**	**0.013**	**1**	**−3.65**	**−6.51**	**−0.78**
Chronic bronchitis, %	−0.95	0.41	1	−0.95	−3.24	1.34
ECG abnormal silent, %	−0.23	0.93	1	−0.23	−5.13	4.66
ECG abnormal in heart disease, %	excluded		1			

*p* of coefficient = probabilities. Delta = difference in the levels of risk factors for the estimate of gained or lost years. lcI: low 95% confidence interval; hcI: high 95% confidence interval.

**Table 5:** MLR Model 4 with covariates measured at baseline in 390 men examined at year 31 versus length of survival from year 31 to 61 of follow-up as the dependent variable. R^2^ = 0.24. Significant risk factors in **Bold**.

Risk Factor	Coefficient	*p* of Coefficient	Delta	Gained (+) or Lost (−) Years	lcl	Hcl
Intercept	68.24	---	---	---	---	---
**Age, years**	**−0.57**	**<0.0001**	**5**	**−2.77**	**−3.49**	**−2.06**
Smokers, %	reference	---	---	---	---	---
**Ex-smokers, %**	**2.41**	**0.004**	**1**	**2.41**	**0.76**	**4.06**
**Never smokers, %**	**3.38**	**<0.0001**	**1**	**3.38**	**2.10**	**4.67**
Unhealthy diet, %	reference	---	---	---	---	---
Intermediate diet, %	1.16	0.17	1	1.16	−0.49	2.81
Healthy diet, %	1.21	0.22	1	1.21	-0.74	3.17
Body mass index, kg/m^2^	0.060	0.65	3.5	0.21	−0.70	1.12
Subscapular skinfold, mm	−0.010	0.90	6.0	−0.06	−1.02	0.90
**Systolic blood pressure, mmHg**	**−0.054**	**0.021**	**20**	**−1.09**	**−1.92**	**−0.25**
Heart rate, beats/min	0.052	0.081	13	0.68	−0.08	1.44
Serum cholesterol, mg/dL	−0.006	0.44	40	−0.25	−0.88	0.38
Protein, gr/day	0.019	0.37	25.0	0.47	−0.55	1.48
SAFA, gr/day	−0.014	0.76	12.0	−0.17	−1.25	0.92
MUFA, gr/day	−0.009	0.71	16.0	−0.15	−0.93	0.63
PUFA, gr/day	0.027	0.57	7.0	0.19	−0.45	0.82
Polysaccharides, gr/day	−0.006	0.15	90.0	−0.50	−1.19	0.18
Oligosaccharides, gr/day	0.005	0.66	35.0	0.16	−0.54	0.86
Myocardial infarction, %	excluded	---	---	---	---	---
Heart failure, %	excluded	---	---	---	---	---
Stroke, %	excluded	---	---	---	---	---
Peripheral artery disease, %	1.28	0.82	1	1.28	−9.87	12.44
Cancer, %	excluded	---	---	---	---	---
Diabetes, %	−3.32	0.10	1	−3.32	−7.29	0.66
Chronic bronchitis, %	−1.32	0.35	1	−1.32	−4.07	1.44
ECG abnormal silent, %	−3.19	0.43	1	−3.19	−11.04	4.66
ECG abnormal in heart disease, %	excluded	---	---	---	---	---

*p* of coefficient = probabilities. Delta = difference in the levels of risk factors for the estimate of gained or lost years. lcI: low 95% confidence interval; hcI: high 95% confidence interval.

**Table 6:** MLR Model 5 with covariates measured at year 31 in 390 men examined at year 31 versus length of survival from year 31 to 61 of follow-up as the dependent variable. R^2^ = 0.32. Significant risk factors in **Bold**.

Risk Factor	Coefficient	*p* of Coefficient	Delta	Gained (+) or Lost (−) Years	lcl	hcl
Intercept	50.84	---	---	---	---	---
**Age, years**	**−0.53**	**<0.0001**	**5**	**−2.59**	**−3.29**	**−1.89**
Smokers, %	reference	---	---	---	---	---
**Ex-smokers, %**	**2.23**	**0.007**	**1**	**2.23**	**0.62**	**3.84**
**Never smokers, %**	**3.10**	**0.001**	**1**	**3.10**	**1.23**	**4.97**
Unhealthy diet, %	Reference	---	---	---	---	---
Intermediate diet, %	1.48	0.065	1	1.48	−0.09	3.06
**Healthy diet, %**	**2.11**	**0.005**	**1**	**2.11**	**0.65**	**3.57**
Body mass index, kg/m^2^	−0.014	0.90	3.5	−0.05	−0.81	0.71
Subscapular skinfold, mm	0.024	0.68	6.0	0.15	−0.55	0.84
Systolic blood pressure, mmHg	−0.017	0.26	20	−0.33	−0.90	0.24
Heart rate, beats/min	−0.030	0.16	13	−0.39	−0.92	0.15
Serum cholesterol, mg/dL	0.004	0.54	40	0.17	−0.37	0.71
Protein, gr/day	0.047	0.061	25.0	1.16	−0.05	2.37
SAFA, gr/day	−0.008	0.88	12.0	−0.09	−1.33	1.15
MUFA, gr/day	−0.030	0.25	16.0	−0.48	−1.31	0.34
PUFA, gr/day	0.014	0.80	7.0	0.10	−0.65	0.84
Polysaccharides, gr/day	−0.002	0.68	90.0	−0.22	−1.27	0.84
Oligosaccharides, gr/day	0.0009	0.92	35.0	0.03	−0.62	0.68
Myocardial infarction, %	−1.069	0.16	1	−1.07	−2.57	0.43
Heart failure, %	0.41	0.66	1	0.41	−1.41	2.23
**Stroke, %**	**−2.70**	**0.004**	**1**	**−2.70**	**−4.53**	**−0.87**
**Peripheral artery disease, %**	**−2.26**	**0.002**	**1**	**−2.26**	**−3.67**	**−0.85**
Cancer, %	0.19	0.87	1	0.19	−1.99	2.36
**Diabetes, %**	**−1.94**	**0.020**	**1**	**−1.94**	**−3.58**	**−0.31**
Chronic bronchitis, %	−0.28	0.71	1	−0.28	−1.73	1.17
**ECG abnormal silent, %**	**−3.25**	**0.001**	**1**	**−3.25**	**−5.19**	**−1.31**
ECG abnormal in heart disease, %	−2.13	0.095	1	−2.13	−4.61	0.36

*p* of coefficient = probabilities. Delta = difference in the levels of risk factors for the estimate of gained or lost years. lcI: low 95% confidence interval; hcI: high 95% confidence interval.

**Table 7:** Summary of findings of MLR findings reported in Table 2, Table 3, Table 4, Table 5 and Table 6.

Risk Factors	Year 0	Year 0	Year 0	Year 0	Year 31
Follow-Up	Year 0–61	Year 0–31	Year 31–61	Year 31–61	Year 31–61
Exposed	All Men	All Men	Alive Year 31	Examined Year 31	Examined Year 31
Age	inverse	inverse	inverse	inverse	inverse
Smokers	reference	reference	reference	reference	reference
Ex-smokers	---	---	direct	direct	direct
Never smokers	direct	direct	direct	direct	direct
Unhealthy diet	reference	reference	reference	reference	reference
Intermediate diet	---	---	---	---	---
Healthy diet	direct	direct	---	---	direct
Body mass index	---	---	---	---	---
Subscapular skinfold	---	direct	---	---	---
Systolic blood pressure	inverse	inverse	---	inverse	---
Heart rate	inverse	inverse	---	---	---
Serum cholesterol	inverse	inverse	inverse	---	---
Protein	---	---	---	---	---
SAFA	inverse	inverse	---	---	---
MUFA	---	---	---	---	---
PUFA	direct	direct	---	---	---
Polysaccharides	---	---	---	---	---
Oligosaccharides	---	---	---	---	---
Myocardial infarction	---	---	excluded	---	---
Heart failure	---	---	excluded	excluded	---
Stroke	inverse	inverse	excluded	excluded	inverse
Peripheral artery disease	---	---	excluded	---	inverse
Cancer	inverse	inverse	excluded	excluded	---
Diabetes	inverse	---	inverse	---	inverse
Chronic bronchitis	inverse	inverse	---	---	---
ECG abnormal silent	---	---	---	---	inverse
ECG abnormal in heart disease	---	---	excluded	excluded	---

Excluded because the prevalence of the condition was 0.

**Table 8:** Estimated survival from multiple linear regressions using a choice of potentially modifiable factors. Good and bad risk profiles were based on the addition or subtraction of 1 standard deviation of the relevant continuous variables. Yes–no choices were made for discrete variables.

	Estimated Survival From Year 0 to Year 61			Estimated Survival From Year 31 to Year 61		
Risk Factors	Mean Risk Profile	Good Risk Profile	Bad Risk Profile	Mean Risk Profile	Good Risk Profile	Bad Risk Profile
Age, years	49.1	49.1	49.1	77.4	77.4	77.4
Smokers, %	0.62	0	1	0.16	0	1
Ex smokers, %	0.14	0	0	0.62	0	0
Never smokers, %	0.24	1	0	0.22	1	0
Unhealthy diet	0.33	0	1	0.33	0	1
Intermediate diet	0.33	0	0	0.33	0	0
Healthy diet	0.33	1	0	0.33	1	0
Systolic blood pressure, mmHg	144	123	165	161	141	181
Heart rate, beats/min	71	58	84	70	57	83
Serum cholesterol, mg/dL	202	161	243	208	167	249
SAFA, gr/day	29	17	41	21	12	30
Estimated survival, years	26.4	32.6	19.0	10.1	12.9	6.3
Gained (+) or lost (−) years	---	+6.2	−7.4	---	+2.8	−3.8

## Data Availability

The data and computing codes are not available for replication because the original data are not publicly available, although the Board of Directors of the Study may evaluate specific requests for dedicated analyses.
